# Fine-Scale Biogeography and the Inference of Ecological Interactions Among Neutrophilic Iron-Oxidizing Zetaproteobacteria as Determined by a Rule-Based Microbial Network

**DOI:** 10.3389/fmicb.2019.02389

**Published:** 2019-10-25

**Authors:** Katherine Duchinski, Craig L. Moyer, Kevin Hager, Heather Fullerton

**Affiliations:** ^1^Department of Biology, College of Charleston, Charleston, SC, United States; ^2^Department of Biology, Western Washington University, Bellingham, WA, United States

**Keywords:** Mariana Arc and back-arc, Lō‘ihi Seamount, hydrothermal vents, community structure, rule-based network, Zetaproteobacteria

## Abstract

Hydrothermal vents, such as those at Lō‘ihi Seamount and the Mariana Arc and back-arc, release iron required to support life from the Earth’s crust. In these ecosystems, bacteria and archaea can oxidize the released iron and therefore play an important role in the biogeochemical cycles of essential nutrients. These organisms often form microbial mats, and the primary producers in these communities can support diverse higher trophic levels. One such class of bacteria are the Zetaproteobacteria. This class of bacteria oxidize iron and commonly produce extracellular iron oxyhydroxide matrices that provide architecture to the microbial mats, so they are considered foundational members of the community and ecosystem engineers. Zetaproteobacteria are responsible for the majority of iron-oxidation in circumneutral, marine, low-oxygen environments. To study the composition of these communities, microbial mats were collected using a biomat sampler, which allows for fine-scale collection of microbial mats. DNA was then extracted and amplified for analysis of the SSU rRNA gene. After quality control and filtering, the SSU rRNA genes from Mariana Arc and Lō‘ihi Seamount microbial mat communities were compared pairwise to determine which site exhibits a greater microbial diversity and how much community overlap exists between the two sites. In-depth analysis was performed with the rule-based microbial network (RMN) algorithm, which identified a possible competitive relationship across oligotypes of a cosmopolitan Zetaproteobacteria operational taxonomic unit (OTU). This result demonstrated the ecological relevance of oligotypes, or fine-scale OTU variants. The oligotype distributions of the cosmopolitan ZetaOTUs varied greatly across the Pacific Ocean. The competitive relationship between dominant oligotypes at Lō‘ihi Seamount and the Mariana Arc and back-arc may be driving their differential distributions across the two regions and may result in species divergence within a cosmopolitan ZetaOTU. This implementation of the RMN algorithm can both predict directional relationships within a community and provide insight to the level at which evolution is occurring across ecosystems.

## Introduction

Microbes are responsible for the recycling of essential macronutrients such as iron, nitrogen, and carbon, which allow life to continue sustainably. These microbes directly impact biogeochemical cycles and can influence multicellular organisms through trophic-level relationships. One classical example of this type of interaction is the production of bioavailable nitrogen by free-living or symbiotic diazotrophs (Hayatsu et al., 2008). Hydrothermal vents are unique ecosystems that release a number of reduced chemicals into the surrounding environment, which in turn support diverse microbial communities. At these habitats, in the absence of photosynthesis, but in the presence of oxygen, autotrophic microorganisms derive energy via the oxidation of reduced chemicals. These microbes then energetically support additional trophic levels more so than the surrounding ocean floor, making for some of the most biologically productive regions of the deep-sea (Jørgensen and Boetius, 2007). Additionally, hydrothermal vents can be separated by large geographic distance, but are still inhabited by similar microbial communities (McAllister et al., 2011; Hager et al., 2017). In these chemosynthetic ecosystems microbial metabolism directly supports diverse animal communities which help define the limits of survival and the elementary requirements of life (Shank, 2010). Therefore, microbial ecology is immensely important to all life on Earth, and this field of study has advanced through the advent of genomic and bioinformatics technologies, now including network analysis techniques.

Analysis of microbial communities using the small subunit (SSU) rRNA gene has been instrumental in advancing microbial ecology by allowing researchers to identify many taxa from an environmental sample (Woese, 1987; Bálint et al., 2016). Bacteria are frequently delineated into operational taxonomic units (OTUs); a definition which is often based on 97% sequence similarity, because the SSU rRNA gene is highly conserved, small variations in the gene could indicate large evolutionary distance (Janda and Abbott, 2007). Popular software packages for analyzing SSU rRNA gene sequences, such as mothur (Schloss et al., 2009) and QIIME (Caporaso et al., 2010), are capable of processing high-throughput sequences for OTU analysis. Further delineation of the OTUs can be performed through oligotyping, where small changes in the conserved SSU rRNA gene are used to indicate important ecological variation (Eren et al., 2013). This approach has previously been applied to refine biogeographic patterns that OTU-based analysis alone could not (Scott et al., 2017). Oligotypes, or clusters of variants with the same short sequence in high-entropy regions of the SSU rRNA gene, can therefore be used as a more sensitive metric for indicators of biogeography.

An understanding of the biogeography of an ecologically significant population across a diverse array of microbial communities helps explain overarching evolutionary relationships (Flores et al., 2012; Mino et al., 2013; Meyer et al., 2018). At hydrothermal vents, evidence of allopatric speciation has been observed in both bacteria and archaea, even though there is also evidence of dispersal (Price et al., 2015; Mino et al., 2017). To better understand factors that influence specific microbial species and their biogeography, the OTU and their abundances is often used (Wong et al., 2015; Westcott and Schloss, 2017; Knight et al., 2018). The relationships between abundance and biotic factors, such as the presence of other taxa, and abiotic factors such as geographical location, temperature, or macronutrient concentration, may be considered. However, microbial biogeography patterns are particularly challenging to discern due to under-sampling of rich communities (Meyer et al., 2018) and the use of higher level taxonomic classifications (Eren et al., 2013). It is also unclear whether genetic variants within an OTU exhibit distinct biogeographical patterns or interact ecologically. Oligotyping, which identifies genetic variants, paired with network analysis could help elucidate the scale of interaction and the biotic factors influencing evolution in hydrothermal vents. Although oligotyping has previously aided biogeographic analysis of hydrothermal vents, the relevance of oligotype discrimination in ecological interactions has not been investigated (Scott et al., 2017). Ecological interactions are defined by their effects on each party: beneficial, harmful, or neutral (Faust and Raes, 2012). An interaction that benefits one group and harms the other is called parasitism. Interactions with no consequences for one group but the other experiences positive or negative effects are called commensalism and amensalism, respectively. Competitive relationships harm both parties; mutualistic or cooperative relationships benefit both. This study aimed to determine if oligotypes of cosmopolitan Zetaproteobacteria OTUs compete, cooperate, or are geographically isolated.

Two hydrothermal vent regions of interest are Lō‘ihi Seamount and of the Mariana Arc and back-arc. Lō‘ihi Seamount is the youngest seamount of the Hawaiian Island chain and actively emits Fe(II) and CO_2_ rich hydrothermal vent effluent, which supports extensive microbial mats (Sakai et al., 1987; Emerson and Moyer, 2010). In contrast, the Mariana Arc and back-arc contain a variety of hydrothermal vent ecosystems due to volcanic activity at a tectonic plate convergence (Kato et al., 2009a; Huber et al., 2010; Trembath-Reichert et al., 2019). These vent sites collectively host microbial mats with the highest documented biodiversity of any known hydrothermal vent habitat (Emerson and Moyer, 2010; Hager et al., 2017).

At both the Mariana region and Lō‘ihi Seamount, Zetaproteobacteria are found in high abundance and form thick microbial iron mats (Fullerton et al., 2017; Hager et al., 2017; Scott et al., 2017). Though recently described, Zetaproteobacteria have been detected in hydrothermal borehole fluids from the Southern Mariana Trough (Kato et al., 2009b), at diffuse vents of in the South Tonga Arc (Forget et al., 2010), in continental subsurface waters (Emerson et al., 2016) and near-shore estuaries (McBeth et al., 2013; Chiu et al., 2017) and intertidal zones (McAllister et al., 2015). Zetaproteobacteria are marine, neutrophilic, microaerophilic iron-oxidizing bacteria that commonly produce extracellular iron oxyhydroxide structures that make up the iron mats (Emerson et al., 2007; Chan et al., 2011). These oxyhydroxides are composed of polysaccharides in a stalks or sheath formation, which become thicker and more iron-encrusted with age (Chan et al., 2011; Fleming et al., 2013). At both Lō‘ihi Seamount and Mariana vent sites stalks have been observed (Chan et al., 2016; Makita et al., 2016). This mat architecture then provides nutrients and is critical in the construction of this habitat for other bacteria, making the Zetaproteobacteria ecosystem engineers at high-iron hydrothermal vent ecosystems (Emerson, 2012; Chan et al., 2016). Furthermore, these iron oxyhydroxides are sensitive to environmental conditions and have potential to serve as indicators of ecosystem health and geochemical conditions at the time of their formation (Konhauser et al., 2011; Krepski et al., 2013). Iron-oxidizing bacteria have further been credited with a significant contribution to the formation of large banded iron formations during the late Archaean and early Proterozoic eras (Konhauser et al., 2011; Chan et al., 2016; Emerson, 2016). Because they live in circumneutral, low-oxygen, marine environments, Zetaproteobacteria are responsible for the majority of iron-oxidation and likely have been throughout much of Earth’s history (Emerson et al., 2010; Hedrich et al., 2011; Field et al., 2016; Beam et al., 2018; Rouxel et al., 2018).

The biogeography of Zetaproteobacteria across iron mats in two geographically distinct hydrothermal vent regions can help define the key interactions in these respective communities. Microbial network construction can elucidate inter-genus, inter-OTU, and intra-OTU relationships of Zetaproteobacteria. A simple approach to constructing networks is co-occurrence, or correlation, of pairs of organisms over multiple samples (Faust and Raes, 2012; Song et al., 2014). Rule-based approaches can infer the strength and directionality of interactions among nodes and may better approximate biological relationships (Xu et al., 2002; Tsai et al., 2015). The recent rule-based microbial network (RMN) algorithm has discovered regulatory relationships among organisms present in microbial communities in the intestinal tract of human infants (Tsai et al., 2015). The RMN algorithm assumes bacterial relative abundance behaves according to the relative abundances of competitor and cooperator OTUs. It tests every permutation of OTU triplets; does OTU “x” behave according to the model if we assume it has a competitive relationship with OTU “y” and a mutualistic relationship with OTU “z”? Triplets that conform to the model are selected for the network. The resulting network represents candidate competitive, mutualistic, and other ecological interactions within the community. However, no software using the RMN algorithm is currently available.

To further elucidate the biogeographic patterns of Zetaproteobacteria, samples collected from hydrothermal vents sites of Lō‘ihi Seamount and the Mariana Arc and back-arc were sequenced at the SSU rRNA level. To perform RMN analysis in this study, an original implementation of the algorithm was created and made available as an open-source program. The implemented RMN algorithm is a command line program that can construct a network directly from mothur or oligotyping native output files. Here, the focus was on the Zetaproteobacteria sequences at the oligotype and OTU levels. This analysis showed differing oligotype distributions between OTUs at Lō‘ihi and Mariana sites and hints at ongoing niche adaptation.

## Materials and Methods

### Sample Collection and Processing

Microbial mat samples from Lō‘ihi Seamount were collected in March 2013 as described in Jesser et al. (2015), except for sample PV340SS which was collected in September 1997 by scoop sampler. Samples from the Mariana Arc and back-arc were collected in December 2014 as described in Hager et al. (2017). Except for PV340SS, all microbial mat samples were collected with the biomat syringe (BMS) sampler (Breier et al., 2012). Once samples were onboard, they were stored at −80°C. DNA was extracted and stored as previously described (Jesser et al., 2015; Hager et al., 2017).

### DNA Sequencing and Sequence Processing

**FIGURE 1:**
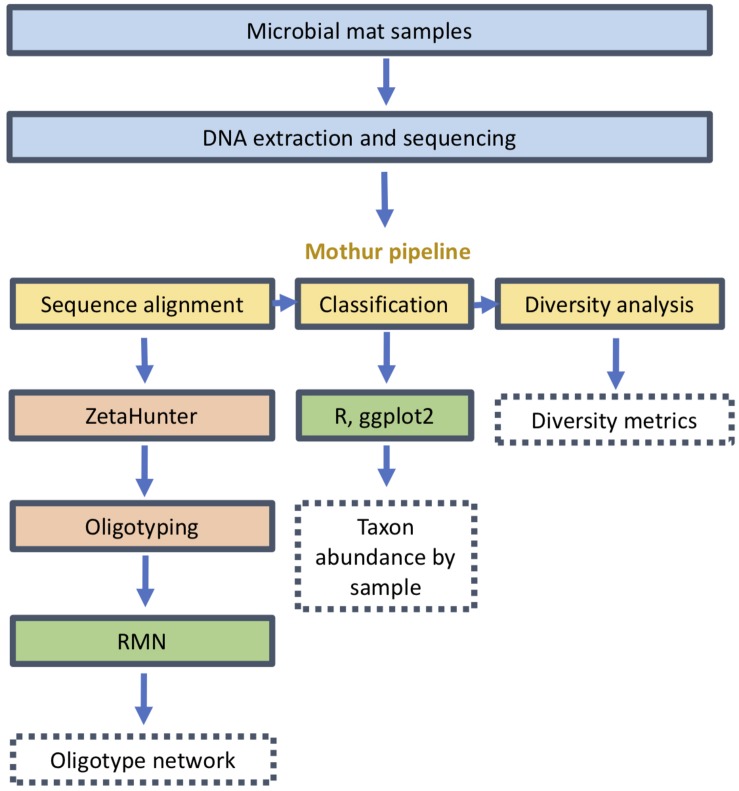
Analysis pipeline from sample collection and sequencing through oligotyping and network inference.

All DNA was extracted as described in previous studies (Jesser et al., 2015; Hager et al., 2017). The extracted DNA was amplified by PCR using bacterial primers 340F and 784R as described by Klindworth et al. (2012) targeting the V3–V4 variable region of the SSU rRNA gene. The resulting amplicons from Lō‘ihi Seamount were processed and sequenced using a MiSeq (Illumina, San Diego, CA) as previously described by Hager et al. (2017). These were processed through the mothur pipeline as previously described (Figure 1; Schloss et al., 2009; Kozich et al., 2013). The reads were trimmed of primers and filtered to remove low-quality and chimeric sequences; the remainder were assigned to OTUs of 97% genetic similarity. Alpha diversity, including observed OTUs, Good’s coverage, Chao1 index, Shannon diversity, inverse Simpson and Shannon evenness, were calculated using mothur (Schloss et al., 2009). Sequences from the class Zetaproteobacteria were isolated and assigned to canonical Zetaproteobacterial OTUs (ZetaOTUs) in the SILVA reference database (version 123) with ZetaHunter (McAllister et al., 2018). Relative abundances of genera and diversity metrics were also calculated through mothur and normalized to the lowest sequencing outcome.

### Oligotyping

The resulting output from mothur was further processed by oligotyping to assess fine-scale differences within the most abundant ZetaOTUs at both Lō‘ihi and the Mariana sites (Eren et al., 2013). The mothur output was transformed into the correct format by a previously developed shell script (mothur2oligo)^1^. Oligotypes were based on the two highest-entropy loci in the V3–V4 region of the SSU ribosomal gene. No additional constraints on minimum abundance or number of samples were imposed. Oligotypes were not resolved to purity due to the high level of diversity of this gene within OTUs. The standard output files containing relative abundances of the resulting oligotypes were used in the network analysis.

### Rule-Based Microbial Network Analysis

To infer microbial ecological relationships, the RMN algorithm was implemented in Python 3.6 as previously described (Tsai et al., 2015). Using the data from Koenig et al. (2011), this implementation was able to recreate the results described in Tsai et al. (2015) (data not shown). The RMN program is executable from the command line and available at https://github.com/kduchinski/RMN. It is compatible with native mothur and oligotyping output such that the program can read relative abundance data in the relabund or matrix_percents.txt files, respectively. Relationships among oligotypes and genera were inferred using the RMN algorithm.

The relative abundances of common taxa and oligotypes at the various sample sites were visualized in R (version 3.3.3). Our ZetaOTU2 oligotype distribution was constructed using the ggplot2 library and the standard oligotyping output was created using the ggplot2 library and the standard oligotyping output. The ecological interaction RMNs were visualized with our novel command line program through the networkx and matplotlib Python packages.

## Results and Discussion

### Bacterial Diversity and Community Structure

A total of 29 samples of microbial mats collected, with 14 samples from Lō‘ihi and 15 from the Mariana Arc and back-arc. All samples, except one from Lō‘ihi Seamount (PV340SS) were collected with a fine-scale syringe sampler (Breier et al., 2012). Paired-end reads were assembled resulting in 15,660,318 contigs with an average length of 420 bp covering the V3–V4 region of the SSU rRNA gene. After chimera checking, quality filtering and removal of sequences that could not be classified as bacteria, a total of 6,955,333 contigs remained representing 69,526 OTUs at the 97% similarity of the SSU rRNA gene.

The observed OTUs varied across all sites (Table 1) ranging from 230 at Lō‘ihi Marker 11 (PV340SS) to 14,173 at NW Eifuku Marker 124 (799D3). Only two samples (673BM1C123456 and 801X345) had a Good’s Coverage of less than 0.98, whereas three samples (676BM1C34, 674BM2D12456, and PV340SS) had a Good’s Coverage of 1.0. Higher values for Chao1 richness estimator, non-parametric Shannon diversity and Inverse Simpson were observed for all samples from FeMO deep and Snail. All these samples were described as flocculant iron mat and the Snail sample had an Mn outer crust. Samples from Pohaku, Champagne, and Jet Vents, showed lowest values for the non-parametric Shannon diversity, Inverse Simpson, and Shannon Evenness (Table 1).

**TABLE 1:** Sample location details and diversity metrics.

**Sample**	**Vent field**	**Site**	**OTUs observed**	**Good’s coverage**	**Chao1 index**	**Shannon diversity^∗^**	**Inverse simpson**	**Shannon evenness**
672BM1B12345	Lō‘ihi	Hiolo North (Mkr 31)	4,604	0.99	7,889	4.8	14.8	0.56
673BM1B123	Lō‘ihi	FeMO Deep	9,149	0.99	14,939	6.4	128.8	0.70
673BM1B456	Lō‘ihi	FeMO Deep	8,751	0.98	15,217	6.8	184.7	0.74
673BM1C123456	Lō‘ihi	FeMO Deep	8,456	0.96	15,823	6.9	173.2	0.76
674BM1A2356	Lō‘ihi	Hiolo North (Mkr 39)	2,767	0.99	5,930	4.1	14.4	0.51
674BM1B123	Lō‘ihi	Hiolo North (Mkr 39)	3,927	0.99	9,013	4.0	11.8	0.48
674BM2C126	Lō‘ihi	Pohaku (Mkr 57)	2,143	0.99	4,821	3.0	4.5	0.38
674BM2C345	Lō‘ihi	Pohaku (Mkr 57)	1,784	0.99	4,998	3.3	9.5	0.43
674BM2D12456	Lō‘ihi	Pohaku (Mkr 57)	1,622	1.00	4,713	1.0	1.4	0.13
675BM1A456	Lō‘ihi	Hiolo South (Mkr 34)	1,092	0.99	2,140	3.7	11.9	0.53
675BM2A456	Lō‘ihi	Hiolo South (Mkr 38)	2,130	0.99	4,283	4.1	21.7	0.53
676BM1C34	Lō‘ihi	Hiolo North (Mkr 31)	2,461	1.00	5,658	3.7	16.8	0.47
676BM2A5	Lō‘ihi	Caldera	3,879	0.98	9,383	5.4	57.1	0.65
PV340SS	Lō‘ihi	Jet Vents (Mkr 11)	230	1.00	530	1.8	3.6	0.33
797D156	Snail	Mkr 108	6,298	0.99	10,257	6.0	106.4	0.69
797D234	Snail	Mkr 108	4,863	0.99	8,031	6.0	120.3	0.70
797B12	Urashima	Snap Snap	3,456	0.99	7,755	4.7	36.8	0.58
797B3	Urashima	Snap Snap	4,816	0.99	11,407	4.0	14.5	0.46
797B56	Urashima	Snap Snap	4,130	0.99	8,387	5.2	42.2	0.61
797C34	Urashima	Saipanda Horn	2,558	0.99	4,490	5.0	28.8	0.63
798B123456	NW Eifuku	Champagne	1,850	0.99	6,653	2.2	3.3	0.29
798C346	NW Eifuku	Yellow Cone (Mkr 124)	5,085	0.99	11,822	4.1	16.6	0.47
799B156	NW Eifuku	Yellow Cone (Mkr 124)	6,866	0.99	13,078	5.7	62.5	0.64
799D124	NW Eifuku	Yellow Cone (Mkr 124)	3,836	0.99	7,900	4.7	40.1	0.57
799D3	NW Eifuku	Yellow Cone (Mkr 124)	14,173	0.99	31,580	5.5	55.5	0.57
799D56	NW Eifuku	Yellow Cone (Mkr 124)	2,997	0.99	4,780	5.3	43.0	0.65
800B12456	NW Rota	Olde Iron Slides	7,247	0.99	12,567	5.1	27.2	0.57
801X126	Urashima	Golden Horn (Base)	2,607	0.98	4,952	4.6	15.4	0.57
801X345	Urashima	Golden Horn (middle)	3,015	0.97	8,064	4.7	18.5	0.57

*All metrics calculated with subsampling to the lowest sequencing effort. ^∗^ Non-parametric.*

**FIGURE 2:**
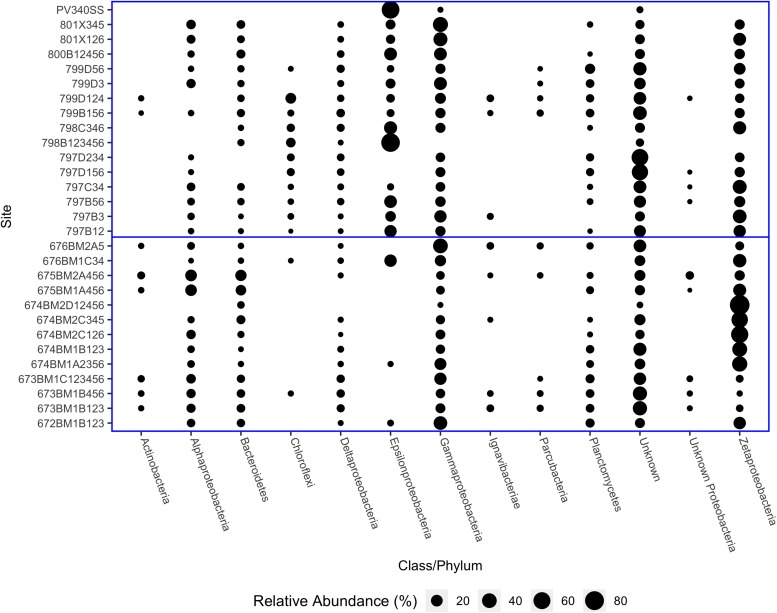
Relative abundances of Proteobacterial classes and other common phyla from Lō‘ihi Seamount and Mariana Arc and backarc samples. Horizontal blue line separates Lō‘ihi samples **(bottom)** from Mariana Arc and backarc samples **(top)**.

Proteobacteria were the most abundant microbial phylum observed at the hydrothermal vents sampled and all classes of Proteobacteria (with the exception of Betaproteobacteria), were identified. Of the Proteobacteria, Zetaproteobacteria were typically the most abundant class and were particularly predominant at Lō‘ihi Seamount microbial mats (Figure 2). Delta and Gammaproteobacteria were present in all samples and varied in abundance from 0.53 to 6.70% (Delta) and 0.12 to 42.81% (Gamma). The abundance of Epsilonproteobacteria was inversely proportional to the abundance of Zetaproteobacteria. Zetaproteobacteria were absent from two samples, one from the Mariana Arc (798B123456) and one from Lō‘ihi Seamount (PV340SS). Epsilonproteobacteria dominated both of these samples, which is unsurprising due to their vent fluid chemistry, which contains high concentrations of dissolved gasses including hydrogen sulfide (H_2_S) and hydrogen (H_2_) (Hager et al., 2017). The dominant genera of Epsilonproteobacteria at most iron mats were *Sulfurovum* and *Sulfurimonas*, with some notable exceptions. At NW Eifuku, Champagne Site (798B123456) was instead dominated by *Thioreductor*, while *Nitratiruptor* and *Caminibacter* were the most abundant genera at Lō‘ihi Seamount Jet Vents (PV340SS). Sample PV340SS was collected shortly after an eruptive event at Lō‘ihi Seamount and so hosted an uncharacteristically high volume of Epsilonproteobacteria. These sites dominated by Epsilonproteobacteria also have some of the lowest observed OTUs, i.e., lowest richness.

Changes in vent chemistry, particularly the concentration of H_2_S, may explain unexpectedly high abundances of Epsilonproteobacteria. Hydrothermal vents are known to release reduced sulfur compounds upon eruption, which would encourage the growth of sulfur-oxidizing Epsilonproteobacteria in normally sulfur-depleted waters of Lō‘ihi (Butterfield et al., 1997). Lō‘ihi Seamount erupted most recently in 1996, and has since returned to pre-eruptive chemistry with elevated levels of Fe(II), and carbon dioxide (CO_2_) (Karl et al., 1989; Glazer and Rouxel, 2009). This location is now relatively dormant, but at the time of microbial mat collection, the T_*max*_ was 196°C. Sample 798B123456 was collected from Champagne vent at NW Eifuku on the Mariana Arc. Champagne vent emits high levels CO_2_, H_2_S and H_2_ and low levels of Fe(II). Few eruptions have been documented at the Mariana Arc and back-arc because underwater eruptions are difficult to observe (Chadwick et al., 2018). However, the relatively high abundances of Epsilonproteobacteria at Mariana iron mats may hint at more recent volcanic activity or greater heterogeneity of the vent effluent.

Bacteriodetes, Chloroflexi, and Planctomycetes were the next most abundant taxa after the Proteobacteria classes. The most abundant class of Bacteroidetes was Flavobacteriia, which was primarily composed of the Flavobacteriaceae family. The Chloroflexi class was divided between members of the Anaerolineaceae and Caldilineaceae families. The dominant Planctomycetes class was Planctomycetia, particularly Planctomyces and other, unclassified Planctomycetaceae. However, many reads in each sample could not be taxonomically classified even at the phylum level (2.39–58.01%). In two Mariana samples, both from Snail (797D234 and 797D156); over 60% of reads from these sites could not be identified. If these unclassified reads do not follow the same distribution as the classified reads, then one or more taxa may be underrepresented (Figure 2).

Zetaproteobacteria ranged in abundance from 0.07 to 89.17%, and *Mariprofundus* spp. were the most abundant genus observed. The predominance of Zetaproteobacteria at the iron mats was consistent with previous studies (Jesser et al., 2015; Fullerton et al., 2017; Hager et al., 2017). The Zetaproteobacteria were especially prevalent at Lō‘ihi sites, and had less Epsilonproteobacteria abundance on average than the Mariana Arc and back-arc (Yamamoto and Takai, 2011), which is correlated to the iron-rich vent effluent observed at Lō‘ihi Seamount (Glazer and Rouxel, 2009). The Pohaku Site at Lō‘ihi Seamount, showed some of the lowest number of observed OTUs, which is consistent with previous studies (Fullerton et al., 2017). The low values for the Shannon evenness can be explained by the dominance of Zetaproteobacteria in the Pohaku, and Epsilonproteobacteria in the Champagne and Jet Vents samples.

### Oligotype Distribution

Though overall bacterial diversity varied widely between Lō‘ihi and Mariana, many of the same Zetaproteobacteria OTUs (ZetaOTUs) were found in both locations with only a few OTUs unique to each hydrothermal vent field. Excluding samples where no Zetaproteobacteria were identified, ZetaOTUs 7, 26, and 36 were only present at Lō‘ihi Seamount and ZetaOTUs 17, 41, and 45 only at the Mariana Arc and back-arc. ZetaOTUs 1, 2, 3, and 4 were the most common, were found at both sites, were present in every sample and together comprised 43.0% of Zetaproteobacteria sequences. This result supports previous findings that ZetaOTUs 3 and 4, as well as 1 and 2, are cosmopolitan (McAllister et al., 2011; Hager et al., 2017). Furthermore, Hager et al. (2017), reported a greater abundance of ZetaOTU 1 compared to ZetaOTU 2 at Mariana and the opposite trend at Lō‘ihi, which was also observed here. ZetaOTUs 9, 11, 18, 23, and 36 have cultured representatives (reviewed in McAllister et al., 2019), each of which were observed, though ZetaOTUs 23 and 36 were found in relatively low abundance (Supplementary Figure S1). ZetaOTU 9 was more abundant at Mariana, though it was present at 8 of 12 Lō‘ihi sites. ZetaOTUs 11 and 18 were found in most iron mats but were more abundant at Lō‘ihi Seamount.

**FIGURE 3:**
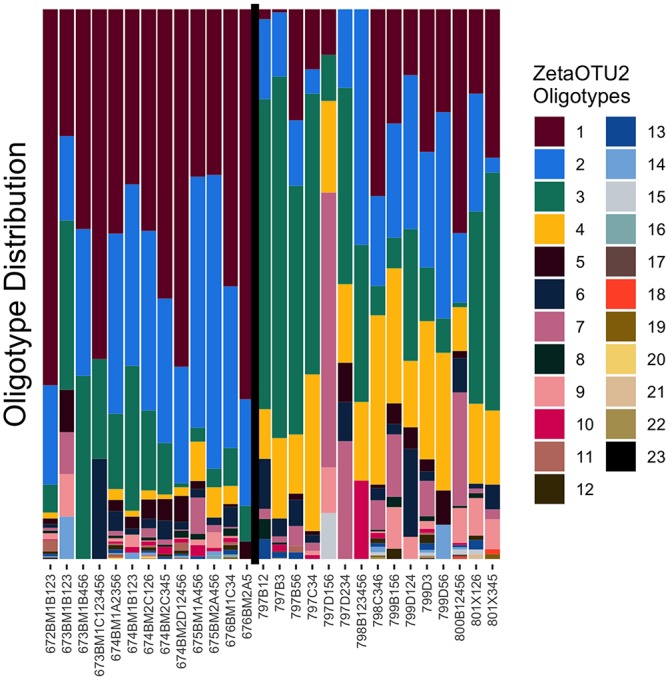
Oligotype distribution of ZetaOTU2. The vertical black line separates the Lō‘ihi Seamount samples **(left)** from the Mariana Arc and backarc samples **(right)**.

Because ZetaOTUs 1, 2, 3, and 4 were the most abundant across both the Mariana and Lō‘ihi sites, they were selected for analysis at the oligotype level, which allows for further delineation within each OTU. Scott et al. (2017) oligotyped Lō‘ihi Seamount and the Mid-Atlantic Ridge but did not detect ZetaOTU 3. Thus, neither oligotyping of the Mariana Arc nor ZetaOTU 3 at Lō‘ihi Seamount have been previously reported. In total 24, 23, 19, and 18 oligotypes were identified from ZetaOTUs 1, 2, 3, and 4, respectively. Analysis of iron mats in from the Pacific Ocean, Mid-Atlantic Ridge and the Arctic Mid-Ocean Ridge suggests that ZetaOTU 2 is globally cosmopolitan (McAllister et al., 2011; Roost et al., 2017; Scott et al., 2017). Though ZetaOTU2 is highly abundant at both the Mariana and Lō‘ihi, oligotyping analysis reveals distinctions in relative abundance between the two sites at a finer-scale than previously detected (Figure 3). Most microbial mats are dominated by 3–4 oligotypes of ZetaOTU2 with a mix of rarer oligotypes making up approximately 10% of the population. However, sample sites with fewer ZetaOTU2 sequences may only host 3–5 oligotypes in total. ZetaOTU2 oligotype 3 is the only one present at every sample site, though oligotypes 1, 2, and 4 are nearly as ubiquitous. Of the ZetaOTU2 at Lō‘ihi Seamount, the most abundant oligotypes are 1, 2, and 3, whereas at the Mariana Arc and back-arc, oligotype 3 is the most highly abundant. ZetaOTU2 oligotype 4 is consistently more abundant than oligotype 1 at all Mariana sites with Zetaproteobacteria. ZetaOTU2 oligotype 6 is also more highly abundant at Mariana than Lō‘ihi and dominates sample 797D156 (Snail). ZetaOTU3 showed a similarly differential distribution of oligotypes, but ZetaOTU3 sequences were rare at most Lō‘ihi Seamount mats. ZetaOTUs 1 and 4 showed less dramatic differences between the two vent regions.

The distributions of oligotypes vary among the sampling sites in each region but are much more consistent among sites of the same region. Lō‘ihi iron mats can be generally characterized by a high ratio of ZetaOTU2 oligotype 1 to oligotype 3 and a low abundance of oligotype 4. Conversely, Mariana iron mats can be characterized by a low ratio of ZetaOTU2 oligotype 1 to oligotype 3 and higher abundance oligotype 4 than what is found in the Lō‘ihi iron mats. Each ZetaOTU was highly entropic even within the oligotype divisions, which indicates that these highly abundant OTUs have highly diversified throughout the Pacific Ocean. Although ZetaOTUs 1, 2, 3, and 4 are found throughout the Pacific, the oligotypes within these OTUs vary greatly, possibly indicating ongoing niche adaptations.

### Rule-Based Interaction Network

**FIGURE 4:**
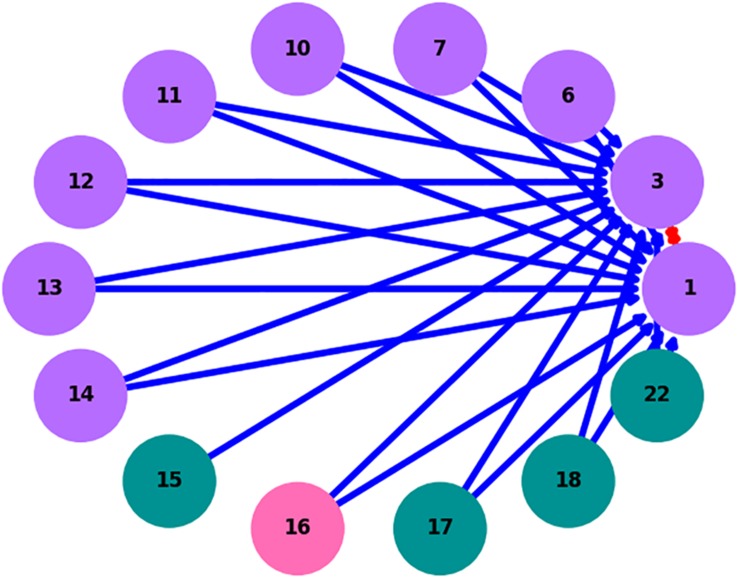
RMN interaction network for ZetaOTU2 oligotypes. Purple nodes denote oligotypes present at both sites, green at Mariana only and pink at Lō‘ihi Seamount only; red edges denote competitive interactions and blue edges denote cooperative interactions.

Previous research on the RMN constructed a microbial community network of the infant gut (Koenig et al., 2011; Tsai et al., 2015). However, no open-source implementation of the RMN algorithm was previously available, therefore the algorithm was implemented in a novel Python program for this study. OTU relative abundances were used in the original implementation of RMN reported by Tsai et al. (2015). This novel program is compatible with relative abundance files as well as oligotyping data and produces a network graphic (Figure 4).

ZetaOTUs 1, 2, 3, and 4 were subjected to RMN analysis following oligotyping to screen for possible ecologically relevant relationships among oligotypes. For ZetaOTUs 1, 3, and 4, no networks were able to be constructed between Lō‘ihi and Mariana microbial mats. The network obtained for ZetaOTU2 (Figure 4) shows a mutual negative interaction between oligotypes 1 and 3, which were the most abundant oligotypes at Lō‘ihi Seamount and the Mariana Arc respectively. It also indicates unilateral positive interactions pointing to these ZetaOTU2 oligotypes from other, less abundant ZetaOTU2 oligotypes in the samples. Most interactions identified by the algorithm are among oligotypes present at both hydrothermal vent fields with only one oligotype specific to Lō‘ihi and two specific to Mariana (at a minimum of 0.1% relative abundance). Networks were successfully constructed for ZetaOTU1 oligotypes at Mariana and ZetaOTU4 oligotypes at Lō‘ihi, but no overarching interactions could be concluded (data not shown). Individual networks at both sites were constructed for ZetaOTU3, but no network was inferred using data from both sites, as the rarity of ZetaOTU3 sequences at Lō‘ihi Seamount likely skewed relative abundance results.

The RMN algorithm highlights candidate ecological interactions based on the relative abundance of OTUs or, in our implementation, oligotypes of an OTU. The advantage of RMN analysis is the power to infer the direction of relationships in a complex of cooperative and competitive interactions. The ability to construct an RMN network from ZetaOTU2 oligotypes suggests that the variance between oligotype distributions in this OTU is biologically significant. By nature of the RMN algorithm, no cooperative interactions affecting an oligotype may be discovered without identifying a competitor for the same oligotype. This feature requires more support for an interaction than pairwise analysis and limits false positive links; the algorithm must provide a potential model of abundance regulation with at least one oligotype that promotes and at least one that impedes the proliferation of the target oligotype. These triplets of OTUs or oligotypes can be better used to construct complex, intertwined networks (Haruta et al., 2009). Thus, we observe many unidirectional cooperative interactions benefiting ZetaOTU2 oligotypes 1 and 3 but cannot conclude any mutualistic relationships between them, and it is statistically unlikely that such relationships exist (Figure 3; Foster and Bell, 2012). As seen in Figure 4, a competitive relationship exists between ZetaOTU2 oligotypes 1 and 3. This competitive interaction is particularly interesting because competition is considered a key driver of evolution (Brown and Wilson, 1956). Coupled with the preference of ZetaOTU2 oligotype 1 for Lō‘ihi and of oligotype 3 for the Mariana, this relationship may result in the evolutionary divergence of these oligotypes. These results indicate that competition may be driving ZetaOTU2 evolution in iron mats at the oligotype level and that this mechanism is consistent at both hydrothermal vent fields despite their geographic distance.

## Conclusion

This study offers insights to the community structure across two similar but distinct hydrothermal vent ecosystems. Microbial iron mats at Lō‘ihi Seamount and the Mariana Arc and back-arc host a diversity of bacterial taxa, but Proteobacteria are particularly populous. Either iron-oxidizing Zetaproteobacteria or sulfur/hydrogen-metabolizing Epsilonproteobacteria are typically the most abundant classes in these communities. Their respective relative abundances are inversely correlated and are likely dependent upon hydrothermal vent fluid chemistry. Different Epsilonproteobacteria genera coexist with Zetaproteobacteria (*Sulfurovum, Sulfurimonas*) than flourish without Zetaproteobacteria (*Thioreductor, Nitratiruptor, Caminibacter*). Flavobacteriaceae and Plantomycetaceae species are also common in microbial mat communities across the Pacific, with Anaerolineaceae and Caldilineaceae populating the Mariana as well as Lō‘ihi Seamount.

The intraspecies genetic diversity and community structure of four cosmopolitan Zetaproteobacteria OTUs: ZetaOTUs 1, 2, 3, and 4, were further investigated. While some regional abundance patterns are observable at the OTU level, oligotyping has illuminated differences in genetic diversity among Mariana and Lō‘ihi hydrothermal vent microbial mat communities at a finer scale. This implementation of the RMN algorithm to infer ecological interactions between the oligotypes of cosmopolitan ZetaOTUs and has discovered a putative competitive relationship between ZetaOTU2 oligotypes 1 and 3. Distinct oligotype distributions and intra-OTU competition suggest niche adaptations driven by competition at the oligotype level. Because of this competition and the differential dominance of oligotypes 1 and 3 across the geographically distant vent field sites, speciation may be dynamically occurring within ZetaOTU2. This method could be used to expand upon the nature of previously observed abundance correlations, to further characterize speciation in microbes and to discover fine-scale genetic differences in biogeography.

## Data Availability Statement

All sequence data are available through the NCBI Sequence Read Archive study number SRP092903 (BioProject: PRJNA352433) for the Mariana Arc and back-arc and Sequence Read Archive study number SRP201760 (BioProject: PRJNA549457) for Lō‘ihi Seamount.

## Author Contributions

KD and HF designed the work. CM, KH, and HF collected the samples. KD, CM, and HF contributed to the bioinformatics, and substantially contributed to the data interpretation, and drafting and revising of the manuscript. KD and HF wrote the manuscript. KH sequenced all the samples and provided valuable insights during revision. KD, KH, CM, and HF responsible for all aspects of this work and approved the final version to be published.

## Conflict of Interest

The authors declare that the research was conducted in the absence of any commercial or financial relationships that could be construed as a potential conflict of interest.

## Footnotes

1 https://github.com/DenefLab/MicrobeMiseq
