# Supplementary figures and images for: Fine-Scale Biogeography and the Inference of Ecological Interactions Among Neutrophilic Iron-Oxidizing Zetaproteobacteria as Determined by a Rule-Based Microbial Network
Source: Front Microbiol. 2019 Oct 25;10:2389. doi: 10.3389/fmicb.2019.02389 (PMC6823593; doi:10.3389/fmicb.2019.02389)

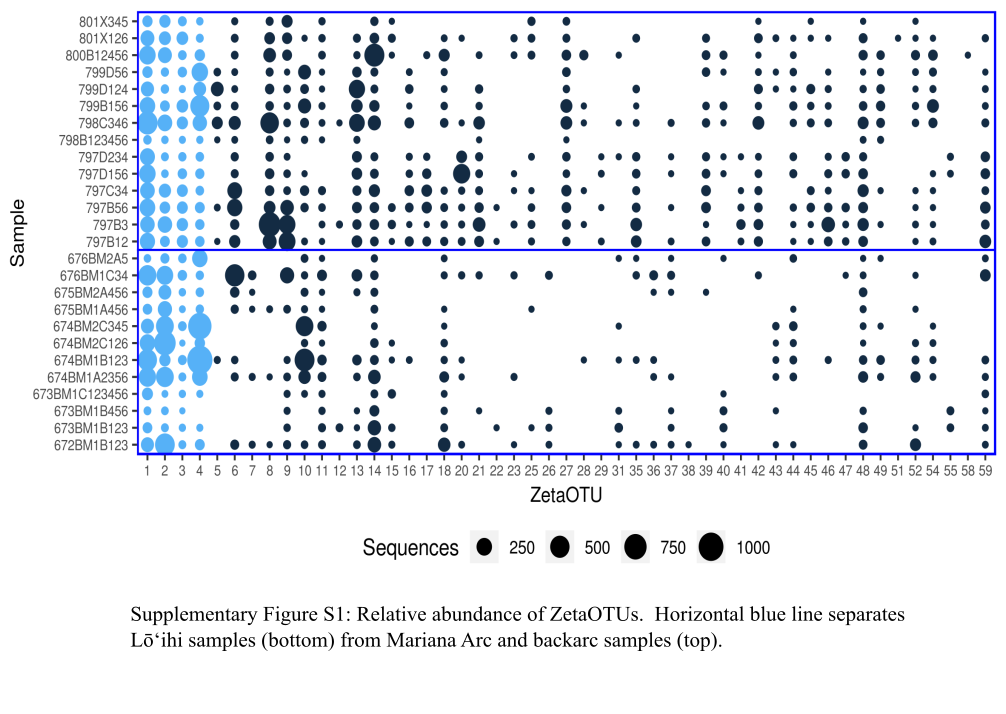

Supplement: Supplementary file 1 [file Image_1.png]
